# Are There CT Imaging Features That Can Distinguish Primary Pulmonary Squamous Cell Carcinoma from Solitary Lung Metastasis of Head and Neck Squamous Cell Carcinoma?

**DOI:** 10.3390/cancers18111703

**Published:** 2026-05-23

**Authors:** Camila Vilela de Oliveira, Corinne C. Liu, Maria Mayoral, Andrew M. Pagano, Eduardo J. Ortiz, Jason Chang, Stephanie Lobaugh, Marinela Capanu, Michelle S. Ginsberg, Andrew J. Plodkowski

**Affiliations:** 1 Department of Radiology, Aura Diagnosticos, Goiania 74063010, GO, Brazil; contact@camilavilela.com; 2 Department of Radiology, Memorial Sloan Kettering Cancer Center, New York, NY 10065, USA; liuc4@mskcc.org (C.C.L.); mayoralm@mskcc.org (M.M.); paganoa@mskcc.org (A.M.P.); ortize7@mskcc.org (E.J.O.); ginsberm@mskcc.org (M.S.G.); 3 Department of Pathology and Laboratory Medicine, Memorial Sloan Kettering Cancer Center, New York, NY 10065, USA; changj2@mskcc.org; 4 Department of Epidemiology and Biostatistics, Memorial Sloan Kettering Cancer Center, New York, NY 10065, USA; lobaughs@mskcc.org (S.L.); capanum@mskcc.org (M.C.)

**Keywords:** lung, solitary pulmonary nodule, squamous cell carcinoma, computed tomography

## Abstract

**Background/Objectives**: Distinguishing primary lung squamous cell carcinoma (PLSCC) from metastatic head and neck squamous cell carcinoma (MHNSCC) to the lungs is challenging for pathologists, especially when patients present with a solitary lung nodule. The purpose of this study was to identify CT imaging features that differ between PLSCC from solitary MHNSCC to the lungs, using next-generation sequencing (NGS) and human papillomavirus in situ hybridization analysis as the gold reference standard. **Methods**: This retrospective, single-institution cross-sectional study included patients with a biopsy-proven PLSCC or solitary MHNSCC from July 2013 to May 2022, who underwent NGS or in situ hybridization, and baseline CT or PET/CT. Each scan was evaluated by at least two radiologists. Nodular, pleural, and ancillary CT features, as well as maximum standardized uptake value (SUVmax) from PET/CTs, were recorded. Associations between imaging features and pathology were examined using either the Wilcoxon rank-sum or Fisher’s exact test. **Results**: In total, 81 patients were included (median 66 years; 64 male); 36/81 (44%) had PLSCC and 45/81 (56%) had MHNSCC. PLSCC was associated with a larger size (median, 3.3–3.6 cm vs. 1.4–1.6 cm, *p* < 0.001), and the presence of post obstructive atelectasis (*p* = 0.002), pleural retraction (*p* < 0.001), pleural tags (*p* = 0.02), and pleural surface involvement (*p* = 0.02). MHNSCC presented as smaller peripheral nodules (*p* = 0.003) with lower SUVmax (*p* = 0.01). **Conclusions**: Several CT imaging features as well as SUVmax from PET/CT were significantly different between PLSCC and solitary MHNSCC and their potential discriminatory ability warrants evaluation in future studies.

## Simple Summary

When someone with head and neck cancer is diagnosed with a single spot in their lung, doctors typically have to ask: is it a new lung cancer or has the original one spread? This is important because the response can affect staging, treatment, and outlook. The authors hope to find out if some aspects on ordinary chest scans, and some on specialized imaging if it is available, can be used to separate the two before expensive or less accessible testing is put to use. When comparing scan findings with more advanced laboratory methods, they found patterns that were more likely to have a new lung cancer and those that were more likely to relate to spread from head and neck cancer. This information could guide researchers to devise more effective imaging-based tools, provide better study designs, and help speed up more informed decision-making in future cancer research.

## 1. Introduction

Distinguishing primary lung squamous cell carcinoma (PLSCC) from metastatic head and neck squamous cell carcinoma (MHNSCC) to the lungs can be challenging for pathologists, as the histopathologic features of these two entities are very similar [1]. Therefore, molecular and human papillomavirus in situ hybridization analyses are valuable additional tools that contribute to this differentiation [2]. In particular, molecular analysis using next-generation sequencing (NGS) is the gold standard method for the differentiation of these lesions, as it is able to depict the clonal relationship between these two entities [3,4]. However, NGS is associated with an increased cost and is not widely available. At institutions where NGS or human papillomavirus in situ hybridization analysis is not readily available, imaging features may need to be relied upon to distinguish primary from metastatic lesions.

The ability to distinguish primary from metastatic lesions is especially important in patients with head and neck squamous cell carcinoma, who can present with either lung metastases or primary lung cancer [5,6]. MHNSCC involves the lung in 70–85% of cases [7,8]. Patients with head and neck squamous carcinoma are also at increased risk of developing PLSCC due to the common risk factor of smoking [9,10]. When these patients present with multiple lung nodules, they have a higher probability of metastases. When they present with a solitary nodule, the question is often raised if a pulmonary lesion is primary or metastatic. The differentiation between primary and metastatic disease is important as it has implications for both staging and management.

CT is a non-invasive imaging modality that is widely available, relatively inexpensive, and is usually performed for follow-up of patients with head and neck squamous cell carcinoma [11,12]. Studies distinguishing PLSCC from MHNSCC in the lung have been performed on the basis of pathology [13,14,15,16], but it is currently not established whether imaging parameters can make this distinction. The objective of this study was to identify CT imaging features that might be used to distinguish primary pulmonary squamous cell carcinoma from solitary lung metastasis of head and neck squamous cell carcinoma, using NGS or HPV in situ hybridization analysis as the reference standard.

## 2. Materials and Methods

### 2.1. Patient Inclusion and Collection of Demographic Information

This retrospective, single-institution cross-sectional study was approved by our institutional review board and the need for informed consent was waived.

Our institution maintains a comprehensive database containing the clinical, pathologic, radiologic, and genomic information of all patients, enabling the identification of patients for this study by applying specific search filters based on the study’s inclusion criteria. An initial search selected patients with biopsy-proven MHNSCC presenting as a solitary lung lesion on a baseline CT or PET/CT, who had histologic assessment with NGS profiling or HPV in situ hybridization analysis, yielding 45 patients consecutively diagnosed between July 2013 and May 2022. Only patients with a solitary pulmonary lesion were included in this study as the presence of multiple lesions is a well-known factor favoring the diagnosis of metastases. We randomly selected 36 patients with PLSCC and histologic assessment with NGS or in situ hybridization from the same database to serve as a control group. The NGS platform used at our institution is a hybrid capture-based method for targeted sequencing of 468 cancer-related genes using solution-phase exon capture and massively parallel next-generation sequencing [17]. Demographic information was obtained from patients’ medical records, including age and biological sex.

### 2.2. Imaging Evaluation

The baseline CT scans of all the included patients (n = 81 patients; 45 with MHNSCC and 36 with PLSCC) were evaluated. Baseline CT scans were mostly standard high-resolution chest CT scans (n = 71/81, 88%); a few were companion CT scans from PET/CTs (n = 10/81, 12%). Baseline CT scans included those performed at our institution as well as those performed at outside institutions. CT scans were distributed among five radiologists with 1–12 years of experience who performed the initial reading while being blinded to pathology results. The second reader was a thoracic specialist with 11 years of experience. In cases of disagreement between the first and second readers, a third reader with 27 years of experience in thoracic imaging evaluated the CT scans to reach a consensus.

CT imaging features that were evaluated included the location, size, contour, and consistency of the solitary pulmonary lesion. Both lesion location in the lung and within the lobe were recorded. The former was characterized as central (contacting a central bronchus to the lobar level), peripheral (beyond the lobar level), or both. Size was measured as the longest dimension on lung window settings in three anatomical planes (axial, coronal, and sagittal). Contour was recorded as lobulated, mass-like consolidation, round/oval, or spiculated. Consistency was characterized as solid (nodular density that obscures underlying pulmonary vessels and airway walls), consolidation (homogeneous hyperdense pulmonary opacity that obscures underlying pulmonary vessels and airway walls), or ground-glass opacity (hazy pulmonary opacity that does not obscure vessels and airway walls) [18].

Additional CT imaging features were also evaluated: invasion of the pleural surface (pleural abutment, pleural nodular thickening/pleural mass, chest wall invasion), and presence of cavitation (pockets of air within the lesion), air bronchograms (visualization of the bronchi lumen with surrounding hyperdense opacity), calcification (any part of the lesion with density greater than 100 Hounsfield units) or post-obstructive atelectasis. Presence of pleural tags (linear extension to the pleura or fissure), pleural retraction, and pleural effusion were also recorded. Lastly, evaluation of mediastinal, hilar, and supraclavicular adenopathy was performed.

Across all PET/CTs (n = 56), the lesion’s maximum standardized uptake value (SUVmax) was obtained. SUVmax was calculated using the following equation, where the maximum-valued pixel within the lesion volume of interest (VOI) was normalized to injection activity (MBq/mL) and patient body weight (kg):

(1) $$SUV=maximum\;activity\;concentration\;in\;the\;VOI\;(\frac{injected\;dose}{patient\;body\;weight})$$

### 2.3. Statistical Analysis

Associations between the varied CT imaging features with PLSCC and MHNSCC diagnosis were examined using the Wilcoxon rank-sum test for continuous variables and Fisher’s exact test for categorical variables. Across all PET/CTs, the association of SUVmax with PLSCC and MHNSCC diagnosis was also examined, using the Wilcoxon rank-sum test. Medians were used in this nonparametric method. All analyses were conducted using R v4.2.2 (R Foundation for Statistical Computing, Vienna, Austria) with tidyverse (v1.3.2) and gtsummary (v1.6.2) packages. The level of significance was set at *p* < 0.05.

## 3. Results

### 3.1. Sample Characteristics

The study sample was composed of 81 patients (median age, 66 years [range, 42–91 years]), 36/81 (44%) who had PLSCC and 45/81 (56%) who had MHNSCC. Most patients were male (64/81, 79%), as seen in Table 1.

### 3.2. Results of CT Imaging Evaluation

**Figure 1:**
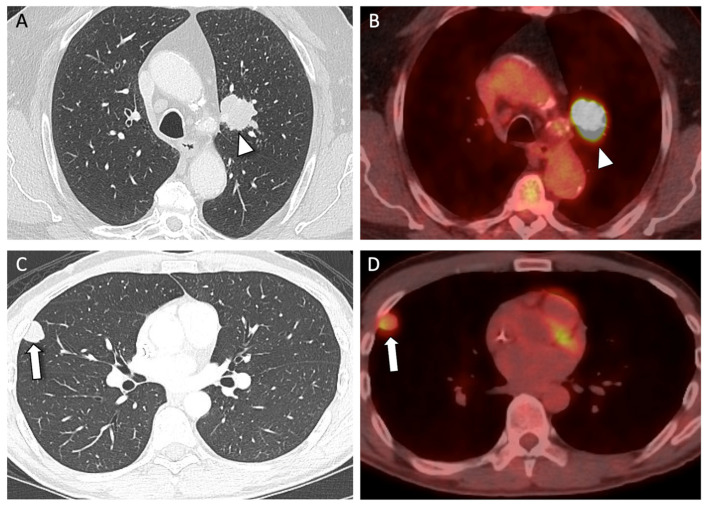
CT axial (**A**,**C**) and PET/CT fused images (**B**,**D**) illustrating the most common location of primary lung squamous cell carcinoma (PLSCC) and metastatic head and neck squamous cell carcinoma (MHNSCC) within the pulmonary lobe and their respective maximum standard uptake values (SUVmax). (**A**,**B**): 66-year-old man with a central left upper lobe primary squamous cell lung carcinoma (arrowhead); SUVmax = 20.9. (**C**,**D**): 45-year-old man with tonsillar squamous cell carcinoma and peripheric right middle lobe lung metastasis (arrow); SUVmax = 3.2.

**Figure 2:**
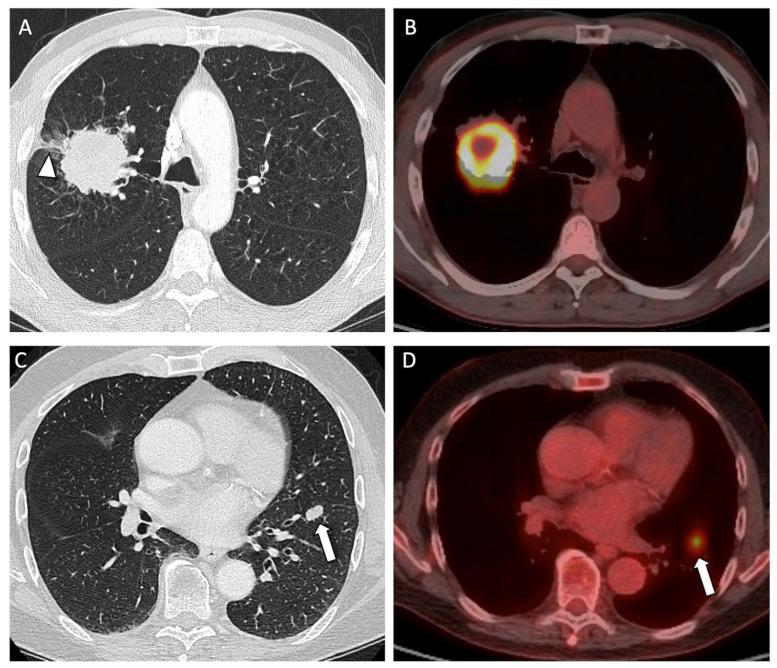
CT axial (**A**,**C**) and PET/CT fused images (**B**,**D**) illustrating the difference in size between primary lung squamous cell carcinoma (PLSCC) and metastatic head and neck squamous cell carcinoma (MHNSCC), with their respective maximum standard uptake values (SUVmax). (**A**,**B**): 58-year-old man with a right upper lobe mass with pleural tag and mild pleural retraction (arrowhead); size = 5.3 × 5.1 cm; SUVmax = 14.7. (**C**,**D**): 70-year-old man with squamous cell carcinoma of the tongue and left tonsil, and left lower lobe lung metastasis (arrow); size = 1.4 × 0.8 cm; SUVmax = 3.2.

The results of CT imaging evaluation are detailed in Table 2. The level of agreement between the first and second readers was 91% (74/81), so that only 9% (7/81) CT images were reviewed by a third reader to reach consensus. Regarding consistency, the vast majority of solitary lesions were classified as solid (78/81, 96%), a few as consolidation (3/81, 4%), and none as ground-glass opacity, with no significant differences between PLSCC and MHNSCC (*p* = 0.58). Regarding location within the lobe, tumor location was significantly associated with MHNSCC status (*p* = 0.003), with a larger proportion of MHNSCC patients with peripheral location compared to PLSCC (87% vs. 56% for MHNSCC and PLSCC, respectively) (Figure 1). PLSCC was significantly larger than MHNSCC in all three anatomical planes, with median sizes of 3.3–3.6 cm and 1.4–1.6 cm, respectively (*p* < 0.001 for all tests) (Figure 2).

**Figure 3:**
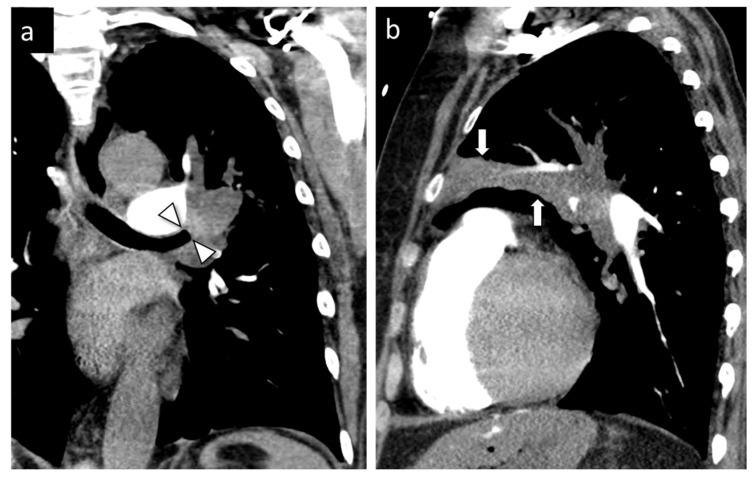
(**a**,**b**): CT coronal (**a**) and sagittal images (**b**) of a 59-year-old woman with left perihilar primary squamous cell lung carcinoma obstructing the left upper lobar bronchus (arrowheads), yielding segmental post obstructive atelectasis (arrows).

Some additional imaging features were also significantly different between PLSCC and MHNSCC. Specifically, the proportion of post-obstructive atelectasis (28% vs. 2%, *p* = 0.002), pleural tags (47% vs. 20%, *p* = 0.02), and pleural retraction (25% vs. 0%, *p* < 0.001) was significantly higher in PLSCC than in MHNSCC (Figure 2A and Figure 3). Only the patients with PLSCC presented with pleural nodular thickening/pleural mass or chest wall invasion (*p* = 0.02).

There was no evidence of a significant difference between groups in terms of distribution of the lesions within the lungs (*p* = 0.26). Lesion contour and presence of cavitation, air bronchograms, calcification, pleural effusion, and thoracic adenopathy (mediastinal, hilar, and supraclavicular) also did not exhibit significant differences.

### 3.3. Results of SUVmax Analysis

Among patients with PET/CT, PLSCC was significantly different and associated with higher SUVmax than MHNSCC (median 11.7 vs. 6.3; *p* = 0.01) (Figure 1 and Figure 2). However, SUVmax values overlapped between groups, and the PET/CT subset was not powered to define a reliable diagnostic cutoff.

## 4. Discussion

To the best of our knowledge, this is the first study to compare CT imaging characteristics between PLSCC and MHNSCC using NGS and HPV in situ hybridization analysis as the reference standard. We found several CT imaging features that showed potential for distinguishing between these entities in patients with solitary lung nodules. Specifically, larger size, presence of post obstructive atelectasis, pleural retraction, and status of the pleural surface were significantly associated with PLSCC. On the other hand, metastatic disease was predominantly characterized by small pulmonary nodules with peripheral distribution. Secondarily, we also found that higher SUV values from PET/CT imaging were significantly associated with PLSCC. These imaging patterns should be interpreted as complementary rather than diagnostic in isolation. Substantial overlap in CT appearance can occur between PLSCC and MHNSCC, and imaging features should therefore be integrated with clinical history, pathology, and molecular testing when available.

It is well known that PLSCC is typically centrally located within the pulmonary lobe and may cause post-obstructive atelectasis when it has endobronchial extension [19]. Additionally, PLSCC has also been shown in previous studies to be larger in size, even reaching more than 4 cm in diameter [20]. Alternatively, pulmonary metastases are typically solid rounded nodules in a peripheral location [21]. Our results are in agreement with previously reported characteristics.

Unfortunately, most cases of PLSCC are diagnosed at an advanced stage, which can explain their larger size at the time of diagnosis [22]. In our study, the fact that PLSCC nodules were significantly associated with more pleural retraction and pleural involvement can also be related to their tendency for being diagnosed at an advanced stage. We also found that PLSCC was significantly associated with pleural tags, in line with previous studies [23,24]. Of note, pleural tags may result from inflammation, fibrosis, or tumor extension.

**Figure 4:**
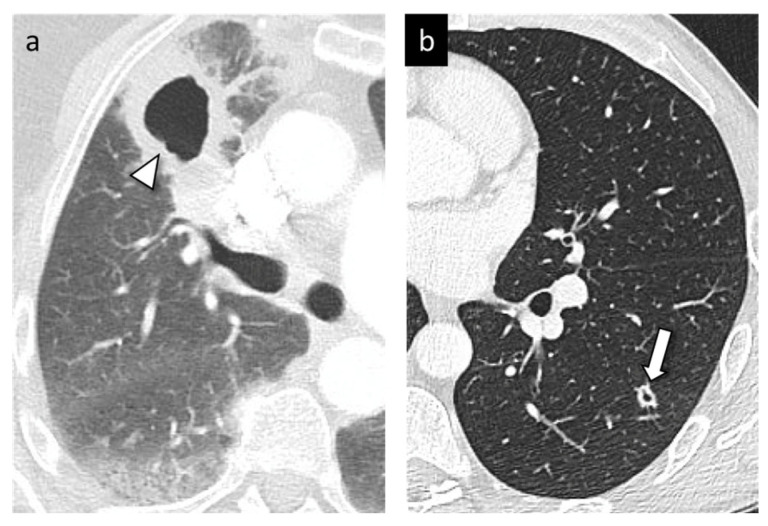
CT axial images of pulmonary nodules with cavitation. (**a**) 60-year-old woman with right upper lobe cavitary mass (arrowhead), consistent with PLSCC. (**b**) 48-year-old man with squamous cell carcinoma of the tongue and left lower lobe cavitary nodule, consistent with metastasis (arrow).

Cavitation is a marker of necrosis and is mostly seen in larger PLSCC and is associated with worse prognosis [25]. While some studies have described this CT imaging feature in both PLSCC and metastatic SCC [20,21], no study has compared the incidence of cavitation in these patients. We noted that the prevalence of cavitation was not significantly different between these two groups in our study (Figure 4).

Although lesion contour has been found to be a valuable CT imaging feature to differentiate primary lung cancer from metastatic nodules in other histologic subtypes [24,26], there was no significant difference pertaining to lesion contour between PLSCC and MHNSCC in our study. All lesions evaluated in our study were classified as either solid or consolidation on CT, corresponding to pathologic findings of sheet-like growth seen in squamous lesions as opposed to spreading along the alveolar wall which is associated with ground-glass opacity on CT [27].

Of note, thoracic nodal metastases are rare in patients with head and neck squamous cell carcinoma, with a reported incidence of mediastinal nodal metastases of 1.1–15.6% [28], but they have a profound impact on prognosis. When evaluating PLSCC, central and larger tumors are at a higher risk of developing nodal metastases [29]. Thoracic adenopathy was found to be more common in patients with PLSCC in our study. However, this difference was not statistically significant, possibly due to our small sample size.

While our study focused on CT findings, many of our patients underwent PET/CT (56/81, 69%), which allowed us to obtain SUVmax data. We found that PLSCC exhibited higher SUVmax values, with a mean of 11.0. Qiu et al. [30] also found that PLSCC is usually associated with higher SUVmax levels when compared to other histologic subtypes, which might also be related to its tendency to present as a larger and more aggressive lesion. To date, no published study in the literature has specifically reported on SUVmax values in MHNSCC to the lungs.

Future studies may also benefit from artificial intelligence and radiomics approaches. Quantitative imaging models could potentially extract features related to lesion shape, margin characteristics, internal texture, peritumoral change, pleural interface, and metabolic heterogeneity that may not be fully captured by qualitative visual assessment. Such approaches could be combined with clinical, pathologic, and molecular data to improve risk stratification. However, larger multicenter cohorts with standardized imaging protocols and external validation would be required before AI- or radiomics-based tools could be applied clinically to distinguish PLSCC from MHNSCC.

Our study has limitations inherent to its retrospective design and small sample size. Although MHNSCC predominantly affects the lungs, MHNSCC presenting as a solitary lesion in the lungs is rare [31], which explains our small number of patients with solitary MHNSCC. Therefore, further prospective and multicenter studies with a larger sample size are needed to confirm our findings and examine their discriminatory role. Another limitation is that CT imaging features were qualitatively assessed and therefore subject to inter-observer variability. Although inter-observer variability was not formally assessed in our study, only 9% (7/81) of the evaluated CT scans required a third reader to reach consensus. If inter-observer variability is an issue, the use of quantitative analysis tools such as radiomics might provide more accurate and reproducible results [32]. Also, the retrospective, single-institution design may introduce selection and referral bias, particularly because the cohort was drawn from a tertiary cancer center with the ability for molecular or HPV-based confirmation.

## 5. Conclusions

In conclusion, our study demonstrates that several CT imaging features as well as SUVmax from PET/CT imaging are associated with PLSCC versus solitary MHNSCC status. As conventional histology-based differentiation is difficult, radiologic imaging features may provide valuable initial staging information and highlight the need for future studies assessing their discriminatory performance.

## Figures and Tables

**Table 1:** Patients’ demographic characteristics.

Characteristic	Total (n = 81)	PLSCC (n = 36; 44%)	MHNSCC (n = 45; 56%)
Age, median (range)	66 (42–91)	71 (49–84)	65 (42–91)
Biological sex, n (%)			
Female	17 (21%)	14 (39%)	3 (7%)
Male	64 (79%)	22 (61%)	42 (93%)

**Table 2:** Imaging features of PLSCC versus solitary MHNSCC.

Characteristic	Total (n = 81)	PLSCC (n = 36) (44%)	Solitary MHNSCC (n = 45) (56%)	*p* Value ^1^
Location in the lung, n (%)				0.26
Lingula	4 (5%)	1 (3%)	3 (7%)	
Left Lower Lobe	16 (20%)	6 (17%)	10 (22%)	
Left Upper Lobe	18 (22%)	9 (25%)	9 (20%)	
Right Lower Lobe	15 (19%)	4 (11%)	11 (24%)	
Right Middle Lobe	8 (10%)	3 (8%)	5 (11%)	
Right Upper Lobe	20 (25%)	13 (36%)	7 (16%)	
Location in the lobe, n (%)				0.003
Central	10 (12%)	6 (17%)	4 (9%)	
Peripheral	59 (73%)	20 (56%)	39 (87%)	
Both	12 (15%)	10 (28%)	2 (4%)	
Size of the nodule (cm), median (range)				
Axial	2.1 (0.7–12.8)	3.3 (0.8–12.8)	1.6 (0.7–6.9)	<0.001
Coronal	2.1 (0.6–10.2)	3.3 (0.7–10.2)	1.5 (0.6–7.6)	<0.001
Sagittal	2.0 (0.7–12.9)	3.6 (0.8–12.9)	1.4 (0.7–7.5)	<0.001
Consistency, n (%)				0.58
Consolidation	3 (4%)	2 (6%)	1 (2%)	
Solid	78 (96%)	34 (94%)	44 (98%)	
Contour, n (%)				0.14
Lobulated	26 (32%)	8 (22%)	18 (40%)	
Mass-like consolidation	4 (5%)	2 (6%)	2 (4%)	
Round/Oval	27 (33%)	11 (31%)	16 (36%)	
Spiculated	24 (30%)	15 (41%)	9 (20%)	
Cavitation, n (%)	10 (12%)	4 (11%)	6 (13%)	>0.99
Air bronchograms, n (%)	8 (10%)	6 (17%)	2 (4%)	0.13
Calcification, n (%)	1 (1%)	1 (3%)	0 (0%)	0.44
Post obstructive atelectasis, n (%)	11 (14%)	10 (28%)	1 (2%)	0.002
Pleural tags, n (%)	26 (32%)	17 (47%)	9 (20%)	0.02
Pleural retraction, n (%)	9 (11%)	9 (25%)	0 (0%)	<0.001
Pleural effusion, n (%)	8 (10%)	4 (11%)	4 (9%)	
Pleural surface involvement, n (%)				0.02
Chest wall invasion	3 (4%)	3 (8%)	0 (0%)	
Nodule thickening/mass	5 (6%)	5 (14%)	0 (0%)	
Tumor abuts the pleura	41 (51%)	19 (53%)	22 (49%)	
Pleural effusion, n (%)	7 (9%)	4 (11%)	3 (7%)	0.69
Ipsilateral	6 (7%)	4 (11%)	2 (4%)	0.40
Contralateral	2 (2%)	0 (0%)	2 (4%)	0.50
Adenopathy, n (%)				
Mediastinal	17 (21%)	10 (28%)	7 (16%)	0.27
Hilar	25 (31%)	15 (42%)	10 (22%)	0.09
Supraclavicular	2 (2%)	2 (6%)	0 (0%)	0.19
SUV max, median (range)	6.9 (1.0–21.8)	11.7 (2.5–20.9)	6.3 (1.0–21.8)	0.01

^1^ *p*-values were obtained using the Wilcoxon rank sum test or Fisher’s exact test. Abbreviations: PLSCC: primary lung squamous cell carcinoma; MHNSCC: metastatic head and neck squamous cell carcinoma.

## Data Availability

The datasets generated during and/or analyzed during the current study are available from the corresponding author on reasonable request.

## Abbreviations

The following abbreviations are used in this manuscript:

MHNSCC	Metastatic head and neck squamous cell carcinoma
NGS	Next-generation sequencing
PLSCC	Primary lung squamous cell carcinoma
SUVmax	Maximum standard uptake value
VOI	Volume of interest
